# CoryneCenter – An online resource for the integrated analysis of corynebacterial genome and transcriptome data

**DOI:** 10.1186/1752-0509-1-55

**Published:** 2007-11-22

**Authors:** Heiko Neuweger, Jan Baumbach, Stefan Albaum, Thomas Bekel, Michael Dondrup, Andrea T Hüser, Jörn Kalinowski, Sebastian Oehm, Alfred Pühler, Sven Rahmann, Jochen Weile, Alexander Goesmann

**Affiliations:** 1 Computational Methods for Emerging Technologies group, Bielefeld University, Bielefeld, Germany; 2 International NRW Graduate School in Bioinformatics and Genome Research, Center for Biotechnology (CeBiTec), Bielefeld, Germany; 3 Bioinformatics Resource Facility, CeBiTec, Bielefeld, Germany; 4 Technology Platform Genomics, CeBiTec, Bielefeld, Germany; 5 DFG Graduiertenkolleg Bioinformatik, Bielefeld University, Bielefeld, Germany; 6 Lehrstuhl für Genetik, Bielefeld University, Bielefeld, Germany; 7 Bioinformatics for High-Throughput Technologies, Dortmund University, Dortmund, Germany

## Abstract

**Background:**

The introduction of high-throughput genome sequencing and post-genome analysis technologies, e.g. DNA microarray approaches, has created the potential to unravel and scrutinize complex gene-regulatory networks on a large scale. The discovery of transcriptional regulatory interactions has become a major topic in modern functional genomics.

**Results:**

To facilitate the analysis of gene-regulatory networks, we have developed CoryneCenter, a web-based resource for the systematic integration and analysis of genome, transcriptome, and gene regulatory information for prokaryotes, especially corynebacteria. For this purpose, we extended and combined the following systems into a common platform: (1) GenDB, an open source genome annotation system, (2) EMMA, a MAGE compliant application for high-throughput transcriptome data storage and analysis, and (3) CoryneRegNet, an ontology-based data warehouse designed to facilitate the reconstruction and analysis of gene regulatory interactions. We demonstrate the potential of CoryneCenter by means of an application example. Using microarray hybridization data, we compare the gene expression of *Corynebacterium glutamicum *under acetate and glucose feeding conditions: Known regulatory networks are confirmed, but moreover CoryneCenter points out additional regulatory interactions.

**Conclusion:**

CoryneCenter provides more than the sum of its parts. Its novel analysis and visualization features significantly simplify the process of obtaining new biological insights into complex regulatory systems. Although the platform currently focusses on corynebacteria, the integrated tools are by no means restricted to these species, and the presented approach offers a general strategy for the analysis and verification of gene regulatory networks. CoryneCenter provides freely accessible projects with the underlying genome annotation, gene expression, and gene regulation data. The system is publicly available at .

## Background

Regulation of gene expression is a highly important mechanism for any organism because it allows the rapid adaptation to changing environmental conditions. Sensing of the surroundings and an appropriate reaction is triggered by complex molecular strategies. The introduction of high-throughput transcriptomics approaches such as DNA microarrays has created the potential to unravel and analyze complex gene-regulatory networks. The study of regulatory interactions has become a major topic in modern functional genomics on a large scale.

To effectively and comprehensively analyze transcriptional regulatory networks, any kind of available and relevant data has to be taken into account and combined. Genome annotations as well as post-genomics data are essential for an in silico analysis of cell behavior.

The CoryneCenter system presented in this article integrates data and analysis features of three different types:

1. Genomic sequence with an up-to-date annotation of regulatory elements, coding sequences, functional RNAs and secondary structures (from the GenDB system [1])

2. Transcriptomics data for the global study of gene expression profiles (from the EMMA database [2])

3. Gene regulatory networks gained from literature, wet lab experiments, and computer predictions (from CoryneRegNet [3-5])

Our contribution concentrates on corynebacteria, organisms relevant in biotechnology, and human medicine. [6] We selected *Corynebacterium glutamicum *as target species for this study, although our approach is not restricted to it.

Genome annotation plays a fundamental role, even and especially in the post genome era. For *Corynebacterium glutamicum*, comprehensive gene annotations have previously been published in [7], along with carefully designed and performed microarray experiments (e.g. in [8-12]).

The challenge of discovering novel regulatory interactions or validating existing networks can be addressed by comparing groups of genes with similar expression profiles that have been identified in microarray experiments with information about the known regulatory network of an organism. Knowledge originating from the genome annotation such as common metabolic pathways is beneficial and fosters the biological interpretation of the results. With the integrated datasets at hand, discrepancies in putative regulatory connections can be easily detected.

In this article, we introduce CoryneCenter, an integrated data analysis platform that connects the above mentioned systems GenDB, EMMA, and CoryneRegNet. Since we follow a federated data integration strategy by using Web Services, the autonomy and up-to-dateness of all systems is granted at any time.

### Existing Systems and Web Services

Heterogeneous experimental and annotation data are stored in numerous life-science databases. Hence, efficient data handling and integration is prevented by a wide range of problems. The major problem, however, is the querying procedure, since it requires detailed semantic knowledge about the content of specific database tables [13].

One approach to overcome these problems is to import data from all sources into a single large database, called a data warehouse. One example is the ONtological inDEXing suite (ONDEX) [14]. Its major benefits are query performance and data integrity. Data integrity is ensured while parsing data from external sources. On the other hand, data updates in any of the remote sources will only be available, if the whole system is updated, which can become very labor-intensive. Furthermore, this approach requires an extensive amount of disk space as well as reliable and fast computing and network infrastructure. An alternative is a federated database system, for example the Comparative Mouse Genomics Centers Consortium (CMGCC) Mouse Federated Database [15] of the National Institute of Health (NIH). Several distributed databases are virtually combined by means of a new database schema on top of the existing ones, which allows a single, integrated, coherent view of all underlying data while the original databases remain accessible as stand-alone services. A query to the federated database is decomposed into sub-queries. These are subsequently submitted to the respective constituent databases and the final result in turn is composed of the separate answers. The benefit of such a system is that one does not have to store and keep up to date all information in a single huge database. On the other hand, depending on the complexity of the federation, considerable expertise might be necessary for designing the federated schema and for querying the database. Whenever the database schema of a source database is changed, the federated schema has to be adjusted as well. Also query performance depends on the single data sources. For the field of genome annotation, a decentralized approach has already been established: The distributed annotation system BioDAS [16] introduces a concept, which allows the integration of annotation data from different servers [17]. BioDAS utilizes an XML-based communication protocol transmitted via a network connection using HTTP.

Other approaches facilitating data integration have a focus on data retrieval and analysis. MyGrid [18], for example, provides an ontology-based mechanism, which can be used by a service provider to register services (e.g., SOAP-based Web Services) and by a service consumer to issue queries for services related to a certain data-type. Subsequent invocations of different services can be combined into re-usable work flows using Taverna [19] and can be made publicly available. MyGrid thus aids in making scientific information and tools accessible to all researchers and helps to provide interchangeable work-flows for different groups. The major advantage of using distributed data and computational resources is that neither data nor computational power need to be hosted or provided by the user. However, if resources change, results will not be reproducible any longer, because there exists no local copy of both data and results.

A prerequisite for distributed Web Service based work flows is a centralized repository that provides information on publicly available Web Services. BioMoby has defined an ontology-based messaging standard [20]. Providers of Web Services can register and offer their services to clients. These consumers can use the BioMoby system to automatically discover and interact with an appropriate service. BioMoby even allows to build data handling pipelines. The major benefits are the definition of a standard in data formats and messaging, the hosting of service providers, as well as answering and managing service requests. Recently, an extension to Taverna was reported to integrate BioMoby services. Furthermore, the data type of a given data set is automatically matched to the input variables of available Web Service functions. Therefore, a list of applicable data processing routines can be presented.

Generally, Web Services can be defined as software interfaces that interact via a network connection using XML-based messages. These either contain queries (function calls) or the corresponding results. While the transfer is usually performed using HTTP, the SOAP protocol can be used to describe the message structure. The description of an entire Web Service is conducted by using the Web Service Description Language (WSDL). Any software that is written in a programming language that offers a SOAP interface can retrieve data directly from the service. Such a program can internally handle all queried data as if the data would be stored in local data structures and memory. Hence, using SOAP based Web Services, the end-user of an integrated platform even does not recognize that the data is obtained from another system. More general information on Web Services and SOAP can be found in [21].

Most interconnections of biological online databases are still realized by using HTML-links to other web pages or by regular, manual downloads and a subsequent integration of the corresponding data. The introduction of Web Services has opened the way to overcome this workaround and to directly integrate, combine, and visualize appropriate data where it is most expedient. The major advantages of Web Services in automatic biological knowledge processing are:

1. No flat files need to be provided by the distributed platforms, so no extra parsers need to be written.

2. All data is stored in the distributed systems and not copied into a local repository. Storage requirements are decreased.

3. No updates or adjustments of the federated database schema are necessary.

4. The repositories do not need to be actively synchronized.

Recently, the access to biomedical Web Services has been published for a growing number of online resources. Some popular examples are: several databases and data analysis services of the EBI [22], the BRENDA database [23], KEGG (Kyoto Encyclopedia of Genes and Genomes) [24], OLS (Ontology Lookup Service) [25], and PathwayExplorer [26].

However, with the existing Web Services no microarray experiments or information on microbial gene regulatory networks can be accessed. Moreover, providing methods for retrieving the necessary data is only the first step towards a successful integrated analysis. The next step, which for the biologist may be more important, is to build a tool on top of this, which allows for convenient data mining and provides suitable visualizations of the integrated data sets. No application is yet known to the authors that uses Web Services to retrieve data for an integrated analysis of microarray experiments and gene regulatory networks coupled to the genome annotation, and provides a user interface for interactively viewing, browsing, and analyzing the data at the same time.

In the following, we introduce GenDB, EMMA, and CoryneRegNet. We describe how we use SOAP to integrate the three data sources to provide the new analysis methods. Subsequently, we illustrate the CoryneCenter software architecture, along with the novel data exchange, visualization and analysis features. Finally, we demonstrate the potential of CoryneCenter by means of an application example. Using microarray hybridization data stored in EMMA, we compare the gene expression of *Corynebacterium glutamicum *under acetate and glucose feeding conditions.

## Construction and content

### GenDB

GenDB is an open source genome annotation system for prokaryotic genomes that has been in productive use for more than six years now and has supported various genome annotation projects, e.g. [7,27,28]. Given a genome sequence, the system integrates numerous tools to perform gene predictions and functional annotations.

For the prediction of coding sequences (CDSs), we first combined the tools Glimmer and Critica [29,30] in Reganor [31] and subsequently integrated Reganor into GenDB. Recently, we developed the gene finder Gismo [32] and also integrated this tool into GenDB. For each detected CDS, a semi-automatic function prediction is performed by using a combination of standard tools: BLAST [33], HMMer, and external repositories, such as SwissProt or InterPro [34]. The resulting observations are assigned to each CDS and further on contribute as basis for the determination of the gene annotation data: gene name, gene product, description, functional category, GO [35] numbers, etc.

Apart from CDSs, further functional elements such as functional RNAs, protein domains and signal peptides are predicted and annotated. The GenDB web interface provides annotation functionality and a multitude of views: contig navigation, a detailed report on each CDS, a region editor for changing gene start positions and the manual creation of sequence features. Comprehensive visualizations, such as virtual 2D gels of proteins or circular chromosomal plots, and pathway maps are included in the interface. For navigating all genes according to their functional classification, the system provides browsing functionality based on the KEGG, COG [36], and GO ontologies. Import and export of annotated genomic sequences are provided at the web interface for FASTA, EMBL and GenBank files.

So far, interactive access to the functionality of GenDB has been granted by the aforementioned web-interface. The new implementation of Web Services into GenDB now allows direct access for remote applications to the data repository and to analysis functions. The availability of up-to-date annotation information of ongoing and finished genome annotation projects is of high importance. GenDB is a functional database system and not a static data repository. Sequence annotations are frequently updated and novel bioinformatics tools are integrated regularly. Web Service technology is well suited to provide data to remote applications in a well structured and platform and programming language independent manner. Although a well documented API (Application Programming Interface) for GenDB already exists, not all research institutes will have the computational power necessary to conduct large scale genome annotation projects. By using Web Services, the results of the computational GenDB pipeline are now publicly accessible for downstream automated processing. Security of the transmitted information is ensured through user authentication and encryption with the Hyper Text Transfer Protocol Sl.

All available Web Services of GenDB have been implemented in Perl using the SOAP::Lite package. We provide detailed and current information on every functional region of a genome, such as CDS, functional RNAs, or transcription factor binding sites. Among other information, the computed GC-content, the exact start and stop positions, and the iso-electric point of the protein can be obtained. Furthermore, curated annotations including associations to COG identifiers or GO terms can be accessed. On a higher level, all genes that are part of the same biochemical pathway can be queried, and all pathways a gene is involved in are returned. This underlying pathway information is computed in the GenDB system based on sequence similarities to genes in the KEGG database.

In the context of CoryneCenter these methods provide valuable and new functionality to CoryneRegNet and EMMA: The user can combine the knowledge about co-regulated genes from CoryneRegNet with observed similar expression profiles from EMMA and the up-to-date functional annotation of the genes, their association to biochemical pathways, as well as their positions on the chromosome, from GenDB to better understand sub-systems of the organism's transcriptional regulation network.

### EMMA

EMMA 2 is a web-based application for the storage and analysis of transcriptomics data from microarrays. It is employed as a centralized transcriptomics repository by several national and international projects, e.g. GenoMik-Plus, Grain Legumes Integrated Project (GLIP) and Marine Genomics Europe (MGE), including projects studying corynebacterial species. An extensible plug-in architecture allows for comprehensive normalization and pre-processing as well as statistical analysis, clustering, and data-mining.

In combination with a Laboratory Information Management System (LIMS) system, EMMA supports MIAME-compliant [37] annotations of the laboratory process and experimental conditions. Furthermore, the import of MAGE-ML compliant files is supported by the system.

The software has an extended three-tier architecture, and similar to GenDB, provides a structured and documented API, making the repository fully compatible with the MAGE-ML standard [38] for microarray data interchange. Using the same core technologies (Perl and SAOP::Lite) as in GenDB, a server module for providing Web Services has been integrated into the presentation layer of the application.

A specific challenge for providing transcriptomics data by using Web Services is the large variety of experiments involving multiple experimental conditions, which may again result in many measured data sets and higher-level analyses. Furthermore, several experiments could co-exist in the repository, while some data should be public and other data has to be kept private. Thus, it is required to simplify access in such a way that a query for expression measurements for a given list of genes under a specific condition results in a single expression value. To accomplish this even in the presence of multiple data, EMMA 2 provides a mechanism to rate the quality of a dataset and to further restrict access of the Web Service server to a curated public sub-set of all data in the repository. As soon as a dataset is made public by the project administrator in EMMA 2, it also becomes accesible through the Web Service.

MAGE-ML identifiers are employed as unique accession keys for experiments, experimental factors, and reporter sequences. A set of utility methods is provided to query for all publicly available identifiers. A standard query is a multi-step process involving an experiment, an experimental factor, and a gene identifier, and returns a single expression value. EMMA 2 is also equipped with an extensible Web Service client, which allows to automatically present external data whenever a nucleotide sequence is displayed. Without further programming, using WSDL service descriptions, the generic client can connect to any Web Service and allows to map different attributes such as reporter nucleotide sequences, sequence names, or species names to the input parameters of remote methods.

For corynebacteria, EMMA 2 now is by default connected to the methods provided by CoryneRegNet to retrieve data on regulatory interactions.

### CoryneRegNet

CoryneRegNet is an ontology-based data warehouse designed to facilitate the annotation and visualization of gene regulatory interactions in prokaryotes. The biological content of CoryneRegNet comprehensively covers transcriptional regulations in the model organism *Escherichia coli K-12 *and pathogenic corynebacterial species of medical importance such as *C. diphtheriae *and *C. jeikeium*. Further data on corynebacteria such as *C. glutamicum *and *C. efficiens*, which are traditionally used in biotechnological fermentation processes, is included. The web interface of CoryneRegNet offers several types of query options. The results of a query are displayed in a table-based style including a visualization of the genetic organization of the respective gene region. Information on DNA binding sites of transcriptional regulators is depicted by sequence logos. The results can also be displayed by several layouters implemented in the graphical user interface GraphVis, allowing, for instance, the visualization of genome-wide network reconstructions and the homology-based inter-species comparison of reconstructed gene regulatory networks.

Using the NuSOAP library for PHP, we have extended CoryneRegNet to provide a SOAP-based Web Service. As for GenDB and EMMA, this enables scientists and software developers to flexibly query the underlying database directly. A query typically returns all regulatory information known about a gene, including literature references. It is also possible to get a list of all organisms in CoryneRegNet and all transcription factor encoding genes of an organism. Furthermore one can pass a list of genes of interest to retrieve all known regulatory interactions of these genes (either their target genes in case of a transcription factor, or transcription factor encoding genes, in case of a target gene), or their operon memberships.

## Utility and discussion

The appropriate combination of data storage and analysis now allows for more easy-to-use web-based user interfaces (no switching between different databases, tools, etc.) on the one hand, but also for better knowledge integration on the other hand. The outcome of a Web Service based connection between GenDB, EMMA and CoryneRegNet is a list of novel data analysis and visualization features, which considering the isolated databases themselves could not have been realized beforehand.

### CoryneCenter architecture

**Figure 1:**
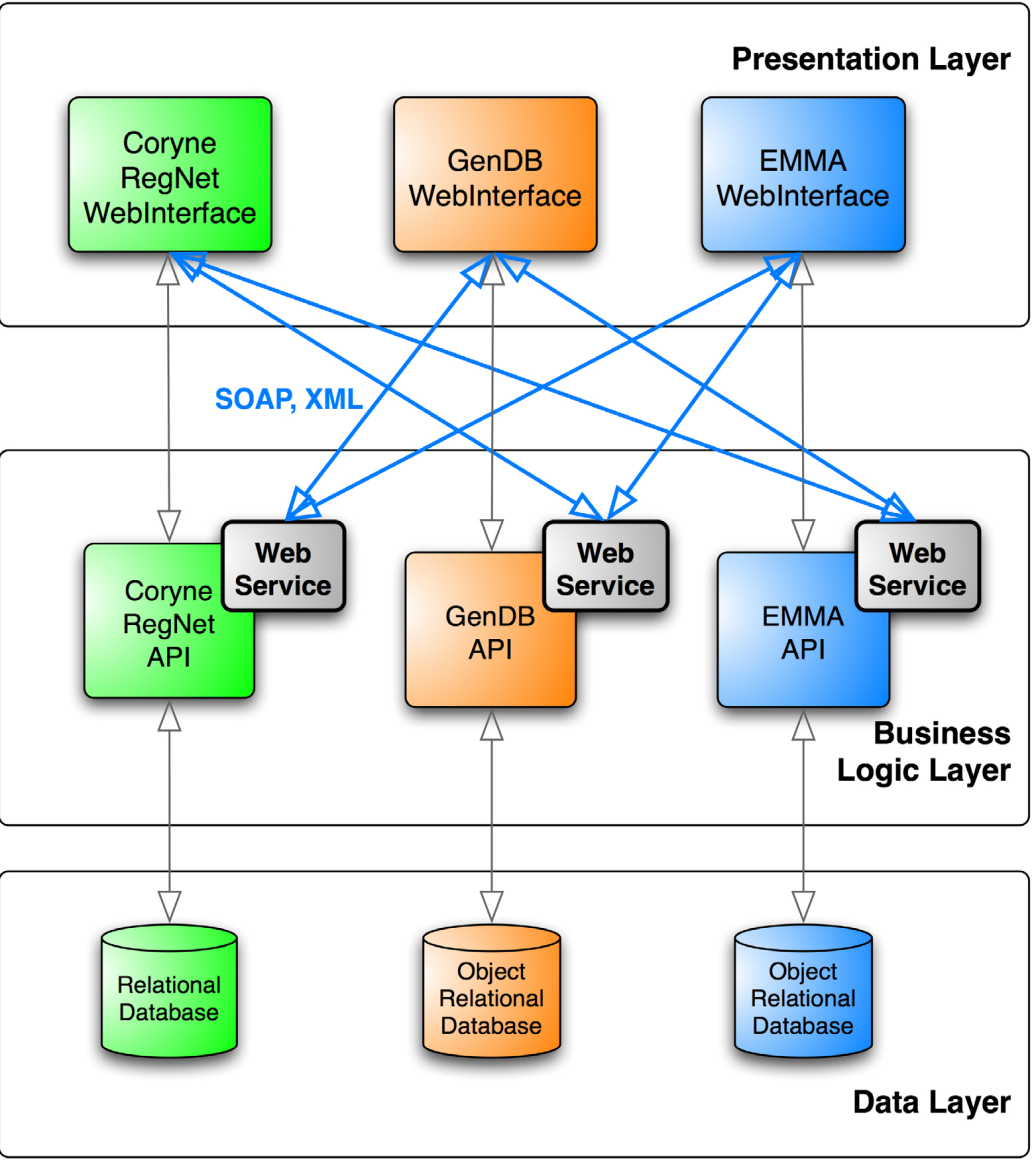
**Architecture of CoryneCenter**. CoryneCenter reflects the three-layer architectures of the connected systems. The respective presentation layers are responsible for the distribution of the web-based user interfaces and rely on the functionality of the underlying business logic layers. Connectivity and data exchange is provided through the newly implemented Web Services. They abstract from the basic data layers and provide structured and easy to integrate functionality to the connected subsystems of CoryneCenter.

The result of our work is the novel platform CoryneCenter that combines the strengths of three established bioinformatics frameworks and seamlessly integrates their data analysis and visualization features. We have integrated SOAP based programming and data exchange interfaces into GenDB, EMMA and CoryneRegNet at all levels of data presentation, analysis and visualization (see Figure 1). Since GenDB and EMMA are implemented in Perl, we provide Web Service servers by means of SOAP::Lite [39] while CoryneRegNet utilizes NuSOAP for PHP [40]. For all of these servers WSDL files have been generated and are available at the CoryneCenter web site. The same freely available software libraries are subsequently used to invoke all three services according to the special purpose (e.g. the PHP based client of CoryneRegNet imports further gene annotation data of the actual visualized gene that is provided by the Perl based Web Service of GenDB).

In the following, we present the novel data analysis and visualization capabilities of CoryneCenter.

### Features

#### GenDB

**Figure 2:**
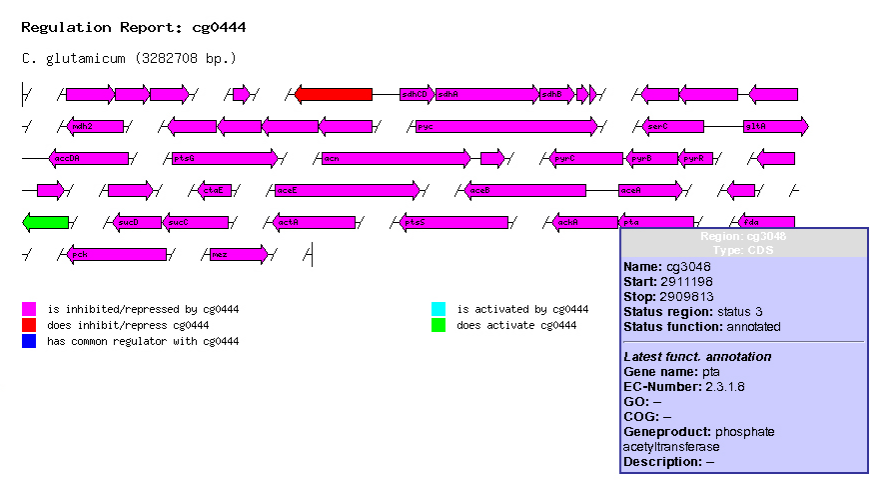
**GenDB integration of CoryneRegNet's web service**. This figure shows a screenshot of the new GenDB gene regulation report presenting the regulatory network of RamB (encoded by *cg0444*) in *C. glutamicum*. Gene regulation information is retrieved via the novel CoryneRegNet Web Service and displayed using different color codes. Here, the user can focus on gene clusters involved in the same regulatory network. Details about the latest annotation of a gene are listed in a tool-tip window. This gives the user a comprehensive and interactive summary of the regulatory elements acting in a specific metabolic pathway.

GenDB now visualizes all known regulatory interactions (retrieved from CoryneRegNet) for a chosen region combined with several links to the corresponding regulators, target genes, and known co-regulated genes (exemplarily shown in Figure 2 for *ramB*, a transcriptional regulator of the MerR family).

The enhanced circular genome plot provides a complete overview of the known regulatory functions of an organism by color-encoding the regulation state of a gene. This provides a fast and comprehensive overview of the regulatory potential of known and novel regulators. Together with the functional annotations that are stored in GenDB and mapped to the interactive visualizations, biologist and genome annotators can now understand regulatory networks in their genomic context.

Additionally, the information on gene regulation is mapped dynamically onto the metabolic pathway maps imported from KEGG. The combination of these novel features and visualizations provides advanced views and analysis methods to the user of CoryneCenter who wants to explore the regulatory potential of a prokaryotic organism. The understanding of gene function and regulatory interactions is enhanced through the efficient and seamless combination of the appropriate data.

#### EMMA

**Figure 3:**
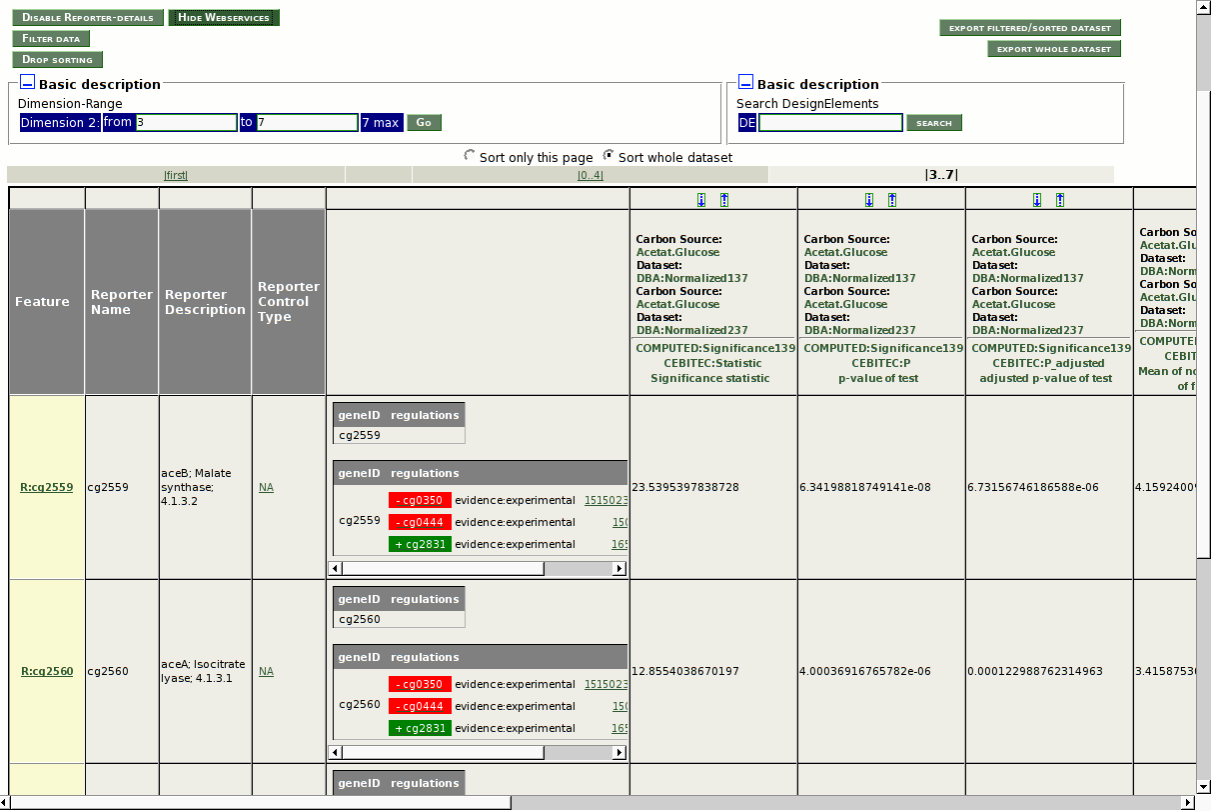
**EMMA integration of CoryneRegNet's web service**. This screenshot exemplarily shows the CoryneRegNet Web Service integration into the EMMA software. Starting from a list of differentially expressed genes, the user can query for gene regulatory information provided by the CoryneRegNet Web Service.

Microarray data visualization and analysis with EMMA profits from the integration of Web Service data. Each biological sequence which is contained in an array design in EMMA can be connected to multiple Web Services as well as conventional sequence databases. The result of an individual Web Service query becomes part of the sequence annotation. It can be displayed whenever a sequence object is visible. This is beneficial when large datasets from microarray experiments are searched. By the integration of CoryneRegNet services, for example, EMMA can now retrieve and depict information on the operon membership of a gene, and the annotated regulatory interactions in which it is involved (see Figure 3). This information can be used to further restrict data tables to genes in a (specific) regulatory network.

#### CoryneRegNet

CoryneRegNet profits on several aspects from the interconnection to GenDB and EMMA. (1) To a gene of interest, more annotation data from GenDB is displayed in the detailed view (EC number, GO number, mapping to KEGG pathways etc.). (2) All target genes of a transcription factor are linked to KEGG to present a list of putative regulated pathways, which allows insights into the general nature of the regulator. (3) Beside this, the built-in graphical network visualization tool GraphVis now features the projection of microarray data extracted from EMMA by altering the size of the concerned nodes according to the expression levels of the represented genes (see the application example in additional file 1). The user can furthermore upload own gene expression data to GraphVis (Excel-file or tab-delimitted flat file) for an integrated analysis with the known gene regulatory network of an organism.

#### Summary

The three software applications GenDB, EMMA and CoryneRegNet now facilitate standardized access to their data sets via the newly implemented Web Service interfaces. Furthermore, the seamless integration into the existing graphical user front-ends of each application provides an added value for comprehensive data analysis on the genome, transcriptome, and gene regulatory level.

Thus, the consistency of gene regulatory predictions can be evaluated and tested using the novel CoryneCenter functionality. As shown in the following application case, the user can test or (in)validate hypotheses that microarray experiments or automated regulatory predictions have generated.

### Application case

#### Dissecting the global transcriptional response in microarray hybridization data by comparing C. glutamicum grown on two different carbon sources

We use CoryneCenter to display transcriptional differences in gene regulatory networks which were detected by microarray analysis of *C. glutamicum *grown with either glucose or acetate as sole source of carbon and energy. The metabolic utilization of glucose and acetate and the cellular adaptation to these different carbon sources are principally different and were subject of intensive investigations in the past (reviewed in [41]).

In *C. glutamicum*, sugars such as glucose are simultaneously taken up and activated by the phosphoenolpyruvate:phosphotransferase system (PTS). The resulting sugar phosphates are metabolized by the glycolysis pathway forming acetyl-Coenzyme A (acetyl-CoA), which then enters the tricarboxylic acid (TCA) cycle.

A different situation emerges when *C. glutamicum *is cultivated on acetate as sole carbon and energy source. Acetate has to be activated by acetate kinase and phosphate acyltransferase. The formed acetyl-CoA enters the TCA cycle. Besides the uptake and activation of acetate, the reactions of the glyoxylate shunt are necessary to replenish the tricarboxylic acid cycle. Theses reactions, catalyzed by isocitrate lyase (ICL) and malate synthase (MS), bypass the TCA cycle to avoid the oxidative decarboxylation steps and finally lead to the formation of the acceptor molecule oxaloacetate from two molecules acetyl-CoA. Oxaloacetate is needed for gluconeogenesis and to keep the TCA cycle running under these conditions [42]. In addition, the reverse operation of glycolysis (gluconeogenesis) is necessary to synthesize essential sugar phosphates. The metabolic and regulatory switch between glucose and acetate consumption in *C. glutamicum *is well-studied. It has been shown that the glyoxylate shunt in *C. glutamicum *is mainly controlled by transcriptional regulation. So far, three different regulatory proteins have been identified that influence transcription of *aceA *and *aceB *by interacting with the upstream regions of these genes encoding the enzymes of the glyoxylate shunt. i) The RamB protein acts as a negative transcriptional regulator on the two genes in presence of glucose [43]. ii) RamA acts as a positive transcriptional regulator in the presence of acetate [44]. iii) GlxR acts as a negative regulator in the presence of cyclic AMP [45]. Of course, all three regulators do not only address *aceA *and *aceB *but act as regulators on overlapping networks including several target genes. All of their known interactions, either experimentally determined from in vitro experiments or predicted, are stored in CoryneRegNet and can be used to dissect the complex data from microarray experiments with the help of CoryneCenter.

In the present study, we analyze the transcriptional stimulon of *C. glutamicum *grown on acetate as sole carbon and energy source. The different influences of each of the three known regulators on their networks will be checked for consistency with the known or predicted regulatory interactions in CoryneRegNet under in vivo conditions. For this purpose we compare the transcriptome of acetate-grown cells to the transcriptome of glucose-grown cells, using microarray hybridization results stored in EMMA.

In CoryneCenter, the data of the microarray experiment can easily be mapped onto regulatory networks. The figure in additional file 1 shows the gene regulatory networks of RamA, RamB and GlxR indicating the relative transcript abundances of the genes encoding the regulatory proteins and of their target genes in acetate-grown *C. glutamicum *cells relative to those from glucose-grown cells. Nodes with green dashed borders indicate enhanced transcript levels, while nodes with red borders describe decreased levels during growth on acetate. The size of the nodes is proportional to the relative differential gene expression (m-value).

In this visualization, the RamA network shows a consistent answer to the stimulus. All target genes except *ramB *showed elevated transcript levels, which correlates to the enhanced transcription of the *ramA *gene. These observations confirm the results of Cramer and coworkers [43], who showed that RamA activates its target genes in the presence of acetate and that the negative auto-regulation of RamA has no influence under this condition. Interestingly, the transcription level of the RamA target gene *ramB *was unaffected (or not detectable) in this experiment. This finding is in contrast to data from the RamB protein quantification by immunoblotting during growth of *C. glutamicum *on different carbon sources, where less RamB protein was found in acetate-grown cells than in glucose-grown cells [46]. In addition, inspection of the RamB target genes showed that most genes are not significantly detected as altered in their transcript levels, which is in accordance to the unchanged *ramB *transcript level. It seems that the regulatory activity of RamB is subdominant in this experiment.

However, the strongly decreased transcript levels of the RamB target genes *ptsS *and *ptsG *encoding sucrose and glucose transport proteins of the PTS system, respectively, could not be explained in this way and point to an additional regulatory network active under acetate or glucose feeding conditions. Recently, the regulator SugR was identified that represses transcription of PTS genes in the absence of sugar-phosphates in *C. glutamicum *[47]. Therefore the detected changes in the transcript level of *ptsG *and *ptsS *are most probably due to a repression by SugR (which is slightly overexpressed) when the cells are grown on acetate. A regulatory effect of GlxR seems not to be dominant, because a consistent de-repression of its regulon was not detected. For the *glxR *gene itself, unchanged transcript levels were expected because the regulatory activity of the protein is thought to be due to an interaction with the second messenger cAMP. It is known that intracellular cAMP levels during growth on acetate are significantly lower than on glucose [45]. This implies that the genes of the GlxR regulon should show enhanced transcript levels. This effect was not consistently detected in the microarray analysis and might mean that the cAMP levels are not different enough to provoke a detectable response in the GlxR regulatory network.

## Conclusion

CoryneCenter was developed as a platform-independent software application that can be easily used via user-friendly web interfaces. As presented in the previous section, CoryneCenter can be effectively applied to the analysis of gene regulatory networks in procaryotes and users can directly benefit from this new kind of data integration. Hypotheses about gene regulation derived by microarray experiments can be easily validated and verified through the integration of transcriptomic, regulatory and genomic datasets.

The use of Web Service technology has been shown to be well suited for the functional integration of the three existing applications GenDB, EMMA and CoryneRegNet. In our approach, the applications' data analysis capabilities are used efficiently, while avoiding the re-implementation of the required functionality in each system. Furthermore, users who are already familiar with one of the tools do not need to learn new interface paradigms and they can enter each application via the new CoryneCenter portal. In addition, new methods for data exchange and communication have been implemented in all three components. These can be used on either side to develop new tools and visualizations. At the same time, up-to-date information (e.g. the latest functional gene annotation) is always presented in each application without any further synchronization. Nevertheless, we still retain a high level of modularity since all Web Service interfaces are implemented as optional plug-in functionality. Thus, all three systems can continuously be developed independent of each other.

However, the integrated CoryneCenter application is more than the sum of its parts as it provides completely new approaches for the generation, visualization, and validation of biological knowledge. In conclusion, CoryneCenter offers a convenient and widely automated way to map expression data onto known or predicted regulatory network interactions. By inspecting the results, complex expression patterns can be dissected into individual regulatory networks which in turn can be checked for consistent behavior in a complex experimental setup. Thereby, regulatory hypotheses can be tested and novel regulatory interactions can be inferred.

All Web Services are publicly available and can be consumed easily by any bioinformatics application as long as if it supports a SOAP interface. Documentation and implementation samples are provided at the CoryneCenter web site.

Although the system has been established with an initial focus on corynebacteria, it is not limited to these microbes alone. CoryneRegNet already provides gene regulatory data for *E. coli *and the necessary steps on how to extend the system to cover more prokaryotic organisms have been described in [5]. GenDB and EMMA are both generic frameworks that support project and user based access to publicly available genome annotations and microarray experiments. With the established infrastructure at hand, the analysis of further microbial regulatory networks is a straightforward task.

## Availability and requirements

Project name: CoryneCenter

Project home page: 

Operating system(s): Platform independent

Programming language: PHP, Pearl

License: Academic Free License (AFL)

Any restrictions to use by non-academics: User should contact Heiko.Neuweger@CeBiTec.Uni-Bielefeld.DE.

Comment: Links to EMMA, GenDB and CoryneRegNet are provided, along with links to the locations of the WSDL files. Additionally, sample PHP and Perl scripts are offered that exemplify how to consume the three Web Services. An online tutorial on how CoryneCenter can be used is provided on the web page.

## Authors' contributions

HN, and JB prepared and developed the overall system architecture. HN, and SO developed and implemented the GenDB Web Service; JB developed the CoryneRegNet Web Service; MD, and SA contributed with the EMMA Web Service. JW implemented internal interfaces, compiled the WSDL files, and created the CoryneCenter web page. JK, AH and TB contributed with the application case data, biological interpretations, and figures. SR, JK and AG proposed to interconnect GenDB, EMMA and CoryneRegNet and supervised the whole project. All authors contributed to writing, read and approved the manuscript.

## Supplementary Material

Additional file 1 CoryneRegNet integration of the EMMA web service. The screenshot shows the reconstruction of the gene regulatory networks of RamA, RamB and GlxR and a simultaneous visualization of relative transcript abundances obtained from comparative microarray analysis of *C. glutamicum *grown on either acetate or glucose as sole carbon source. Color code – blue node: gene of the selected regulator (RamA); green nodes and arrows: activators and activating regulatory interactions; red notes and arrows: repressors and repressing regulatory interactions; grey nodes: regulated target genes preceded by a transcription factor binding site; grey boxes: regulated target genes that are part of an operon and not preceded by a transcription factor binding site; red dashed node borders: significantly reduced amount of transcript in the acetate grown culture compared to the glucose grown culture; green dashed node borders: significantly enhanced amount of transcript in the acetate grown culture compared to the glucose grown culture; black bordered nodes: insignificantly altered transcript levels. The size of the nodes is proportional to the relative differential gene expression measured in the microarray experiment (m-value). Click here for file
