# Supplementary figures and images for: CoryneCenter – An online resource for the integrated analysis of corynebacterial genome and transcriptome data
Source: BMC Syst Biol. 2007 Nov 22;1:55. doi: 10.1186/1752-0509-1-55 (PMC2212648; doi:10.1186/1752-0509-1-55)

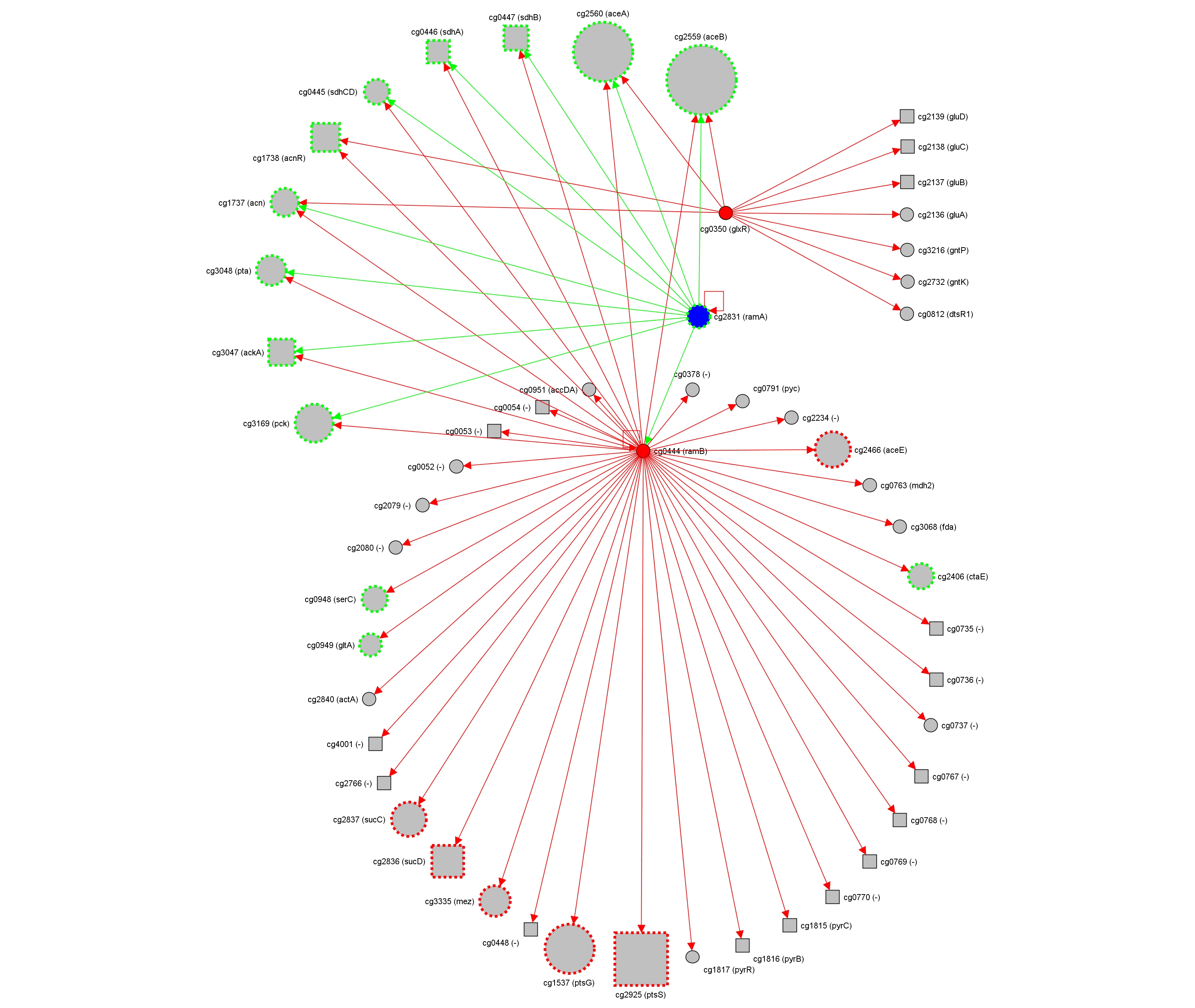

Supplement: Additional file 1 — CoryneRegNet integration of the EMMA web service. The screenshot shows the reconstruction of the gene regulatory networks of RamA, RamB and GlxR and a simultaneous visualization of relative transcript abundances obtained from comparative microarray analysis of C. glutamicum grown on either acetate or glucose as sole carbon source. Color code – blue node: gene of the selected regulator (RamA); green nodes and arrows: activators and activating regulatory interactions; red notes and arrows: repressors and repressing regulatory interactions; grey nodes: regulated target genes preceded by a transcription factor binding site; grey boxes: regulated target genes that are part of an operon and not preceded by a transcription factor binding site; red dashed node borders: significantly reduced amount of transcript in the acetate grown culture compared to the glucose grown culture; green dashed node borders: significantly enhanced amount of transcript in the acetate grown culture compared to the glucose grown culture; black bordered nodes: insignificantly altered transcript levels. The size of the nodes is proportional to the relative differential gene expression measured in the microarray experiment (m-value). [file 1752-0509-1-55-S1.jpeg]
